# The Modification and Performance of a Large Animal Anesthesia Machine (Tafonius^®^) in Order to Deliver Xenon to a Horse

**DOI:** 10.3389/fvets.2017.00162

**Published:** 2017-09-29

**Authors:** Bruna Santangelo, Astrid Robin, Keith Simpson, Julie Potier, Michel Guichardant, Karine Portier

**Affiliations:** ^1^Section of Anesthesiology, Université de Lyon, VetAgro Sup, Marcy l’Etoile, France; ^2^Vetronic Services, Newton Abbot, United Kingdom; ^3^GREAT, Laboratoire Carmen, INSERM U1060, INRA U1235, INSA Lyon, Université Claude Bernard Lyon 1, Villeurbanne, France

**Keywords:** xenon, horses, administration, inhalation, cardiovascular system, recovery, anesthetic machine

## Abstract

**Introduction:**

Xenon, due to its interesting anesthetic properties, could improve the quality of anesthesia protocols in horses despite its high price. This study aimed to modify and test an anesthesia machine capable of delivering xenon to a horse.

**Materials and methods:**

An equine anesthesia machine (Tafonius, Vetronic Services Ltd., UK) was modified by including a T-connector in the valve block to introduce xenon, so that the xenon was pushed into the machine cylinder by the expired gases. A xenon analyzer was connected to the expiratory limb of the patient circuit. The operation of the machine was modeled and experimentally tested for denitrogenation, wash-in, and maintenance phases. The system was considered to consist of two compartments, one being the horse’s lungs, the other being the machine cylinder and circuit. A 15-year-old, 514-kg, healthy gelding horse was anesthetized for 70 min using acepromazine, romifidine, morphine, diazepam, and ketamine. Anesthesia was maintained with xenon and oxygen, co-administered with lidocaine. Ventilation was controlled. Cardiorespiratory variables, expired fraction of xenon (FeXe), blood gases were measured and xenon was detected in plasma. Recovery was unassisted and recorded.

**Results:**

FeXe remained around 65%, using a xenon total volume of 250 L. Five additional boli of ketamine were required to maintain anesthesia. PaO_2_ was 45 ± 1 mmHg. The recovery was calm. Xenon was detected in blood during the entire administration time.

**Conclusion:**

This pilot study describes how to deliver xenon to a horse. Although many technical problems were encountered, their correction could guide future endeavors to study the use of xenon in horses.

## Introduction

Anesthesia in horses is a particularly risky procedure with a mortality rate reaching 1% in healthy patients. Postoperative fractures and myopathy are the most frequent causes of perioperative death in horses (1). Fractures may be caused by anesthetic drug-induced ataxia during recovery. Heavy body mass and lateral or dorsal recumbency limit lung expansion and blood flow to tissues, leading to hypoxemia and/or myopathy. In this regard, it is of prime importance to develop anesthetic drugs that will limit ataxia and maintain sufficient tissue perfusion.

Xenon, a noble gas used in healthy human patients, displays interesting properties (2). It is non-inflammable and non-explosive, has analgesic and hypnotic effects, lack of metabolism, low toxicity, and devoid of teratogenicity. It does not trigger malignant hyperthermia, preserves regional blood flows, and protects organs against ischemic injury. It does not alter respiratory and cardio vascular functions; it has moderate environmental impact (2, 3). Its low blood/gas partition coefficient (0.115) (4) allows fast and smooth induction and recovery and its minimal hemodynamic effects can preserve tissue perfusion.

Xenon is a very rare molecule naturally representing 0.0000086% of the atmosphere’s gases, and it is extracted by cryogenic fractional distillation as a by-product of oxygen purification (5). This makes xenon a very expensive gas, and in human anesthesia its use is four to seven times more expensive than using halogenated inhalant anesthetics (6). However, the development of technologies to purify xenon using adsorption processes (7) could allow an increased supply for its medical use. In addition, availability of xenon may increase due to the fact that automotive industry does use more and more LED technology instead of xenon. This could have the consequence of increasing the quantity of xenon available on the market and, therefore, decreasing its price. Another solution is to set up a breathing system that would avoid wastage of xenon. Such systems already exist in human anesthesia, using the principle of fully closed circuits (8). Similar devices have been experimentally developed in pigs (9).

Xenon has not been studied in equine species; therefore, this study aims to modify a model of large animal anesthesia machine in order to enable the delivery of a measurable amount of xenon to a horse. Theoretical calculations and the clinical application in one equine patient to confirm functioning of the machine are presented.

We hypothesized that our calculation would determine the best method for reaching anesthetic levels of xenon (70% xenon, 30% oxygen) with minimum usage.

## Materials and Methods

### Modification, Modeling, and Experimental Testing of the Anesthesia Machine

**Figure 1:**
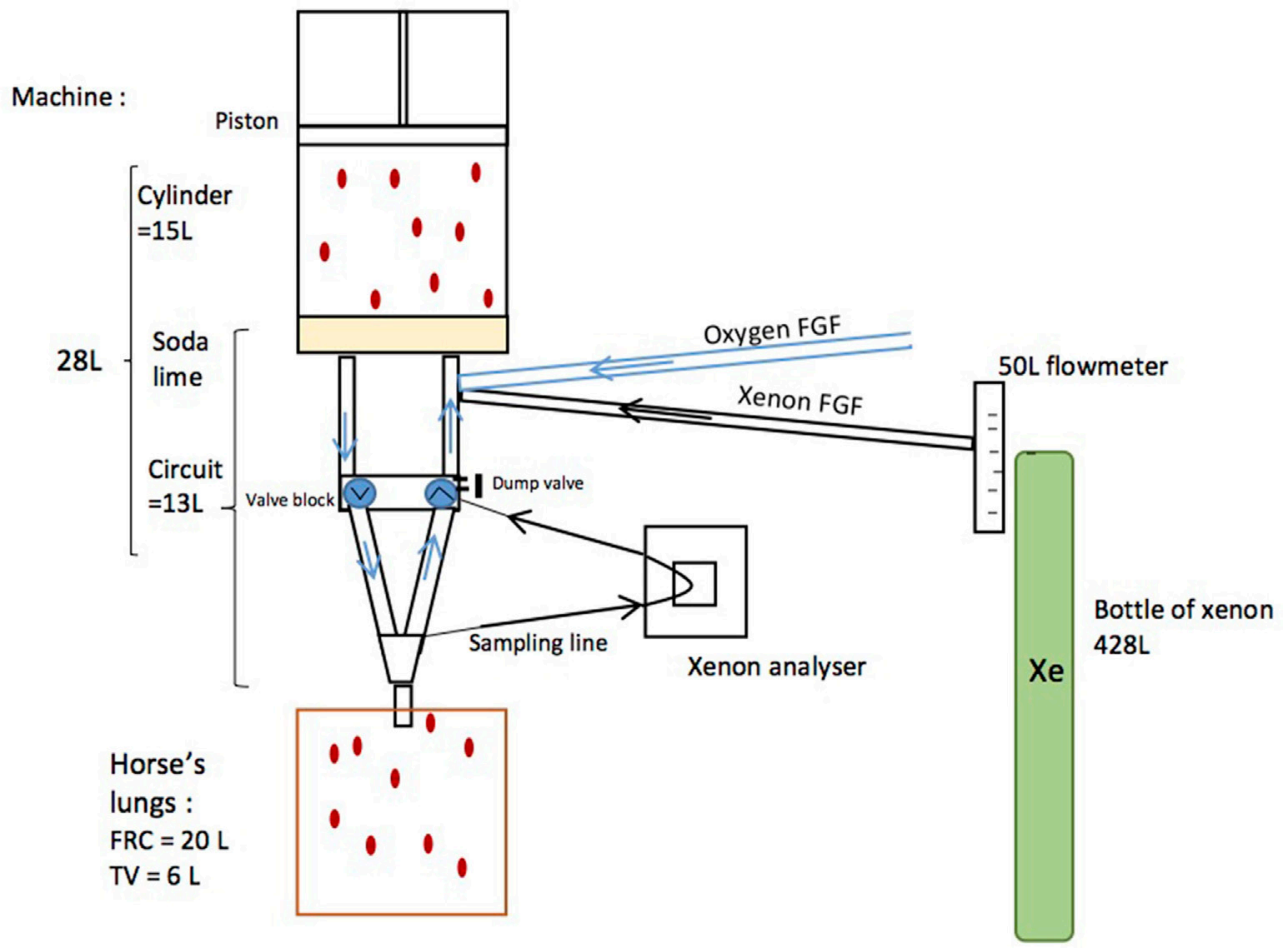
Schematics of a modified Tafonius machine. Xenon was directly taken from a 428-L xenon cylinder connected to a 50-L min^−1^ flow meter and then delivered by a pipe into the bottom of the machine cylinder. A xenon analyzer was connected to the expired limb and sampled expired gases were re-introduced into the bottom of the machine cylinder.

A regular equine anesthesia station (Tafonius T34, Vetronic Services Ltd., Newton Abbot, UK) was modified so as to deliver xenon (Figure 1). Before the experiment, the system was fully checked, cleaned, and resealed by the company (Vetronic Services Ltd., Newton Abbot, UK). The Y-piece was sealed with its cap, and leakage was measured using the electronic leakage calculation mode built in the Tafonius workstation. The modifications included the addition of a T-connector in the valve block for the introduction of xenon (Xenon, Messer, France), so that the xenon flow was pushed into the machine cylinder by expired gases. Xenon (Xe) was directly taken from a 428-L xenon cylinder [*F*, 40 bar (580 psi), 19.5 kg], which was connected to a 50 L min^−1^ flow meter and then delivered by a pipe into the bottom of the main machine cylinder. Tubes on the Y-piece were added to allow gas sampling at the inspired and expired limbs of the patient tubing. A xenon analyzer (K1550, MTL instruments, Kerpen, Germany) was connected to the expired limb only, and the re-introduction of the sampled expired gas into the valve block was done by means of a T-connector. In order to avoid dilution of the system, the automatic addition of oxygen if the machine cylinder volume fell to 0 was disabled.

During the anesthesia and delivery of gases, three periods should be considered: the denitrogenation phase, the wash-in phase, and the maintenance phase. The operation of the machine was modeled and then experimentally tested for each phase using CO_2_ instead of xenon. The xenon analyzer is not gas-specific so CO_2_ will cause a reading to be shown on the screen. In trials prior to the anesthetic, 100% CO_2_ was used as a model for 100% xenon and it was shown that 100% CO_2_ gave a full-scale reading of 28% Xe on the xenon analyzer. Using this relationship, the system could be tested prior to the experiment and still include the integrated xenon analyzer without the obvious expense of using xenon. The xenon analyzer was calibrated for a mixture of oxygen and xenon. In our experiment, a gas mixture of oxygen, xenon, and CO_2_ would be present. The manufacturers of the analyzer advised that the CO_2_ component would be additive at the same scaling factor as with CO_2_ and oxygen. Taking 100% CO_2_ and a reading on the xenon analyzer of 28%, the predicted effect of CO_2_ on the xenon reading is that each 1% of CO_2_ will elevate the xenon reading by 0.28%. This means that without correction, a 60-mmHg (7.9%) end-tidal CO_2_ reading would elevate the xenon reading by 2.2%.

The calculations were repeated until a strategy was devised to minimize the amount of xenon needed to reach the targeted expired fraction of xenon (70% FeXe).

The system was considered to consist of two compartments, one being the horse’s lungs and the other being the machine.

The horse had an estimated FRC of 20 L (10) and was ventilated with a tidal volume (TV) of 6 L. The machine had a cylinder volume of 15 L and a circuit volume (including the intergranular space of soda lime) of 13 L. The machine’s volume was, therefore, 28 L (13 L + 15 L = 28 L) and this was measured before the experiment (Figure 1).

During ventilation a volume of gas equal to the TV was moved from machine to the horse during inspiration and back to the machine during expiration. An active dump valve on the machine limited the machine volume to 28 L, so that any fresh gas added to the system would displace existing mixed gas from the system. A circle system built into the Tafonius machine controlled directional gas movements and ensured gas mixing and CO_2_ removal.

### The Denitrogenation Phase

If the Tafonius machine is prefilled with 100% oxygen then it can be shown (Table 1) that after only six breaths near full mixing of the compartments (machine and horse’s lungs) will have taken place and oxygen levels in both compartments will be near 67%. Knowing that the oxygen consumption of the horse averages 3–5 mL kg^−1^ min^−1^ (11), during this brief phase oxygen needs only to be supplied at a rate greater than the consumption rate of the horse, estimated to be 2.57 L min^−1^ for a 514-kg horse.

**Table 1:** Modeling of denitrogenation.

Breath	New horse O_2_%—FeO_2_	New system O_2_%—FiO_2_
1	39.23	86.98
2	50.25	79.73
3	57.05	75.83
4	61.39	73.89
5	64.27	73.07
6	**66.30**	72.90
7	67.83	73.11
8	69.04	73.52
9	70.08	74.04
10	70.99	74.62
11	71.83	75.23
12	72.61	75.85
13	73.36	76.47
14	74.08	77.08
15	74.77	77.67
16	75.44	78.26
17	76.09	78.83
18	76.72	79.39
19	77.34	79.93
20	77.93	80.46

*The denitrogenation phase assumes a machine prefilled with 100% oxygen, a machine’s volume of 28 L and horse FRC of 20 L. Calculations predicted that with an oxygen flow at 8 L min^−1^ the FeO_2_ reached 67% after six breaths*.

To show why gas reaches 67% after full mixing:

Initial oxygen volume in horse’s lungs FRC = 0.21 × 20 L = 4.2 L.
Oxygen in machine = 1.0 × 28 = 28 L.

Therefore, after connecting the horse to the machine at the end of expiration, mixed gas value is 4.2 L + 28 L = 32.2 L of oxygen in the total volume of 20 + 28 L = 32.2/48 × 100% = 67%.

After this initial minute of mixing, xenon can be introduced.

### The Wash-In Phase

**Figure 2:**
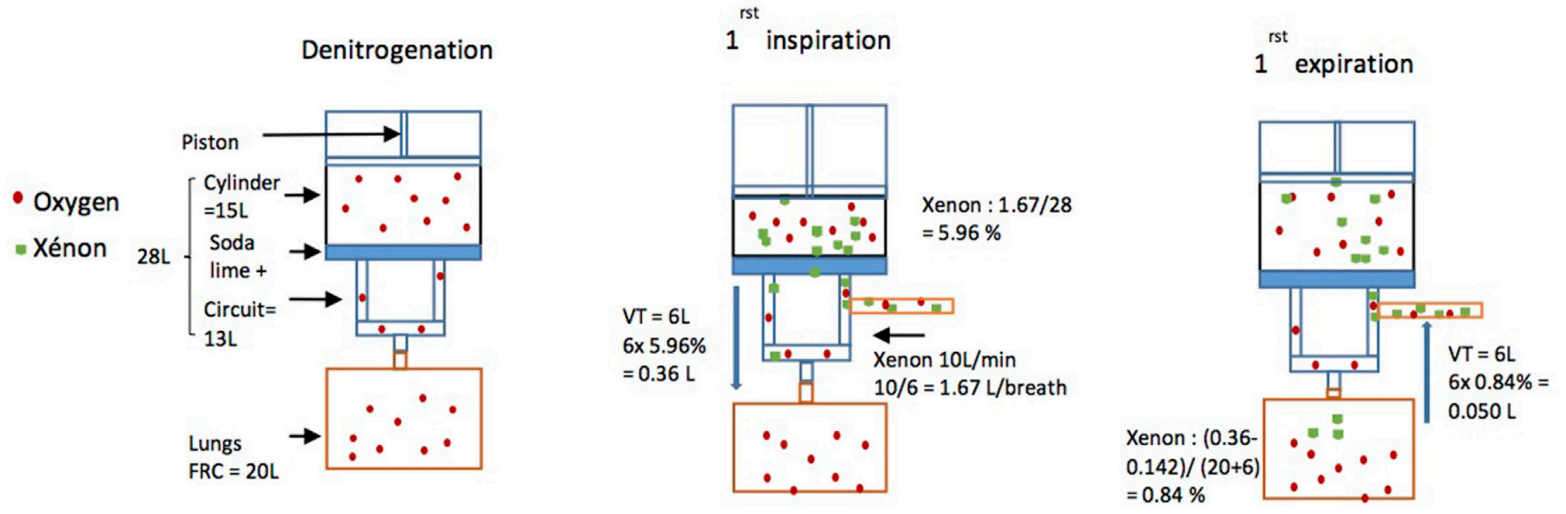
Schematics of the modeling of gas movements. During the denitrogenation phase, xenon flow was turned off. Then xenon flow was started at 10 L min^−1^. As *f*_R_ was six breaths per minute during the experiment 10 L/6 = 1.67 L of xenon were delivered from the bottle into the machine for each breath. Just before the first breath, as the total volume of the machine was 28 L, the total percentage of xenon in the machine was 1.67 L/28 L = 5.96%. So, when the horse breathed xenon for the first time, as the tidal volume (TV) was 6 L, the horse breathed 6 L × 5.96% = 0.36 L of xenon (Vol Xe insp). The horse’s consumption was assumed to be 0.1 L kg^−1^ h^−1^ (= 0.142 L per breath). The new percentage of xenon in the horse’s lungs was then the amount breathed in less the amount consumed, divided by the total volume of the lungs (FRC + TV) so (0.36 − 0.142)/(20 + 6) = 0.84%. Then, as the horse breathed out 6 L containing 0.84% of xenon back into the machine, 6 L × 0.84% = 0.05 L of xenon were expired back into the machine (Vol Xe exp).

Then, a *wash-in phase* was initiated. In order to fill the circuit with 70% xenon and 30% oxygen, the oxygen flow was turned off, while xenon was added until FiO_2_ dropped to 30%. A Microsoft Excel spreadsheet was used to anticipate breath by breath the movements of gas (Figure 2) and determine which xenon flow was the most appropriate.

Due to displacement of mixed gas by incoming gas there is a balance between xenon gas flow rate and time to reach 70% xenon in the horse. Using the spreadsheet model, it was possible to predict that a slight xenon advantage can be gained by higher xenon flow rates compared to lower ones:

For example, at 10 L min^−1^ flow the volume of xenon required to reach 70% is 80 L while at 8 L min^−1^ flow the volume of xenon required to reach 70% is 82.7 L.

On this basis, a flow rate of 10 L min^−1^ was chosen as the preferred delivery rate of xenon in the wash-in phase.

The % Xe in the horse’s lungs at any breath is given by:

Horse % Xe at end expiration (A):

$$\sum_{n\text{=1}}^{n\text{=}b}\frac{\text{Vol Xe insp }(n)\;-\text{ Vol Xe exp }(n)}{\text{FRC}}$$

where *b* is the breath number.

Horse % Xe at end inspiration (B):

$$\sum_{n\text{=1}}^{n\text{=}b}\frac{\text{Vol Xe insp (}n-\text{1)}-\text{ Vol Xe exp (}n-\text{1) + Vol Xe insp }(n)}{\text{FRC + TV}}$$

The % and thus volume of Xe in the system that delivers the TV is given by:

Machine % Xe at end expiration (C):

$$\sum_{n\text{=1}}^{n\text{=}b}\frac{\begin{array}{c} \text{Vol added Xe }(n)\;-\text{ Vol Xe transferred to horse }(n)\;-\text{ Vol Xe lost via exhaust }(n) \end{array}}{\text{machine volume}}$$

Vol Xe insp: this is the volume of xenon delivered in the TV and is calculated from the % xenon in the (machine volume) × TV (D).

Vol Xe exp: this is the volume of xenon in the expired breath after the delivered TV has mixed with the horse’s FRC (E).

Vol added Xe: this is the amount of xenon added between breaths and = xenon flow rate × respiratory cycle time (F).

Vol Xe transferred to horse: this is the sum of (D − E).

Vol Xe lost *via* exhaust: this is the volume of xenon lost through the exhaust valve as incoming gas displaces system gas. The total gas volume lost is equal to F (outgoing volume = incoming volume) and the amount of xenon lost is equal to: % xenon at end expiration × (F) = (C) × (F).

The spreadsheet repeatedly calculates the values of D, E, and F for every breath taken passing the calculated values forward to the next breath.

The spreadsheet (Table 2) indicated that it would take 6 min or 38 breaths and required 63 L of xenon to reach a FeXe of 70%.

**Table 2:** Modeling of wash-in phase.

Breath	Horse Xe%—FeXe	Cumulative Xe volume required (L)	Horse O_2_%—FeO_2_	System O_2_%—FiO_2_
1	0.00	1.67	67.00	63.01
2	1.37	3.33	66.08	59.92
3	3.51	5.00	64.66	57.37
4	6.05	6.67	62.98	55.15
5	8.78	8.33	61.17	53.16
6	11.59	10.00	59.32	51.32
7	14.40	11.67	57.47	49.58
8	17.17	13.33	55.65	47.93
9	19.89	15.00	53.87	46.35
10	22.53	16.67	52.14	44.83
11	25.10	18.33	50.45	43.37
12	27.58	20.00	48.82	41.95
13	29.99	21.67	47.23	40.59
14	32.32	23.33	45.70	39.27
15	34.58	25.00	44.21	37.99
16	36.76	26.67	42.78	36.75
17	38.87	28.33	41.39	35.56
18	40.90	30.00	40.04	34.40
19	42.87	31.67	38.74	33.29
20	44.78	33.33	37.48	32.20
21	46.62	35.00	36.26	31.16
22	48.40	36.67	35.09	30.14
23	50.12	38.33	33.95	29.16
24	51.78	40.00	32.84	28.22
25	53.39	41.67	31.77	27.30
26	54.95	43.33	30.74	26.41
27	56.45	45.00	29.74	25.55
28	57.90	46.67	28.78	24.72
29	59.30	48.33	27.84	23.92
30	60.66	50.00	26.94	23.14
31	61.97	51.67	26.06	22.39
32	63.24	53.33	25.21	21.66
33	64.47	55.00	24.39	20.96
34	65.65	56.67	23.60	20.28
35	66.80	58.33	22.83	19.62
36	67.90	60.00	22.09	18.98
37	68.97	61.67	21.37	18.36
**38**	**70.01**	**63.33**	20.68	17.77
39	71.01	65.00	20.01	17.19
40	71.97	66.67	19.36	16.63

*Immediately after the denitrogenation phase, the spreadsheet starts with a FeO_2_ of 67% and a xenon flow of 10 L min^−1^. Calculations used to model gas movements, considering a FRC of 20 L, a tidal volume of 6 L, and *f*_R_ of six breaths min^−1^, indicated that a FeXe reached 70% at breath 38 (6 min) requiring 63 L of xenon*.

When the wash-in phase was initiated, the oxygen flow was turned off, while xenon was added until FiO_2_ dropped to 30%. The calculation showed that inspired oxygen levels would reach 30% in 22 breaths or 3 min and 40 s. This indicates that oxygen should be turned on again around 3 min after the beginning of the wash-in phase to prevent FiO_2_ falling below 30%. This acts to further reduce the inspired xenon concentration and increase the time and volume of xenon required. Modeling showed that starting 1 L min^−1^ of oxygen at breath 22 results in xenon levels reaching 70% after 41 breaths and prevents FiO_2_ falling below 21%. The total amount of xenon required in this case is 72 L.

During the *maintenance phase*, it was decided to manually adjust both xenon and oxygen flows in order to keep the expired xenon level between 60 and 70% and oxygen at 30%. If oxygen concentration dropped below this level, the oxygen flow would be increased until the inspired oxygen fraction reached 30%. In a fully closed circuit, the maintenance of xenon levels requires only to add to the system what is consumed; mainly by storage in fat or by leaks. We assumed that the consumption in horses would be similar to that in humans (0.06–0.1 L kg^−1^ h^−1^) (12). This would require between 31 and 52 L h^−1^ for our 514-kg patient.

### Clinical Trial

The study was performed in accordance with the EUROGUIDE on the accommodation and care of animals used for experimental and other scientific purposes published by the Royal Society of Medicine Press Limited (London, UK). The experimental procedure was approved by the Animal Ethics Committee of VetAgro-Sup (veterinary campus of Lyon), France (no. 1535).

A 15-year-old, 514-kg, healthy gelding horse was included in the study.

Before the trial the horse was fasted for 12 h with free access to water. After clipping and surgical disinfection of the skin, two 14-gauge catheters (Angiocath, Becton Dickinson, UT, USA) were placed in the left and right jugular veins.

Acepromazine 0.04 mg kg^−1^ (Calmivet; Vétoquinol SA; France) was administered intramuscularly 30 min before the induction of general anesthesia. Morphine (Morphine Clorhydrate Aguettant; Aguettant Laboratory; France) at 0.1 mg kg^−1^ and romifidine (Sedivet, Boehringer Ingelheim, Belgium) at 0.06 mg kg^−1^ were administered intravenously (IV) for premedication. 10 min later anesthesia was induced with diazepam (Valium Roche; Roche France) at 0.05 mg kg^−1^ and ketamine (Imalgène 1000, Merial SA, France) at 2.2 mg kg^−1^ IV. A silicone endotracheal tube (inner diameter 26 mm) was introduced into the trachea and the cuff was inflated to ensure a sealed airway. The horse was transferred to the operating room, placed in dorsal recumbency and connected to monitoring equipment. Ringer’s Lactate (Ringer lactate; Laboratoire Aguettant, France) was administered at 5 mL kg^−1^ h^−1^. The endotracheal tube was connected to a circle anesthetic system forming part of the modified anesthetic machine. This system had been prefilled with 100% oxygen. Intermittent positive-pressure ventilation (IPPV) [respiratory frequency (*f*_R_): 6 breaths min^−1^, TV: 6 L, inspiratory time 2.5 s] was started immediately (T0). The horse received a loading dose of lidocaine (1.5 mg kg^−1^ IV; Lurocaine; Vétoquinol, SA) from an automatic infusion pump (Syramed MSP 6000; Arcomed AG, Switzerland) over a period of 20 min (T0 − T20). This was followed by a constant-rate infusion (CRI) of 0.05 mg kg^−1^ min^−1^. Anesthesia was maintained for 70 min with xenon, as described previously. Immediately before transferring the horse to the recovery box lidocaine CRI was stopped and the horse received romifidine (0.02 mg kg^−1^ IV). Once in the recovery room the horse was extubated and oxygen was supplemented *via* a nasal tube. The recovery was unassisted and video recorded.

The depth of anesthesia was recorded every 5 min using clinical signs (presence of palpebral reflexes, nystagmus, and spontaneous movements). The absence of palpebral reflexes, lacrimation, and nystagmus were considered signs of adequate anesthetic depth. If anesthesia was considered insufficient a supplemental dose of ketamine (0.6 mg kg^−1^ IV) was administered.

The following parameters were recorded every 5 min using a pre-calibrated monitor (Solomon system, Vetronic, Newton Abbot, UK): heart rate (HR), end-tidal carbon dioxide (P_E_’CO_2_), inspired, and expired fractions of oxygen (FiO_2_, FeO_2_) and oxygen hemoglobin saturation (SpO_2_), with the probe placed on the tongue. The expired fraction of xenon (FeXe) was measured by a previously calibrated katherometer. Invasive arterial blood pressure (IBP) was measured using a Datex S/5 monitor (Datex-Ohmeda, GE healthcare, Lyon, France) from a 20-Gauge arterial catheter (Insyte-W, Becton Dickinson, UT, USA) placed in the facial artery. The pressure transducer was zeroed against the atmospheric pressure and leveled at the height of the scapulohumeral joint. Dobutamine (Dobutamine Panpharma 250 mg, Panpharma, France) IV was ready to be administered with a syringe driver (Syramed μSP6000, Arcomed, Switzerland) at a starting dose rate of 2 µg kg^−1^ min^−1^ if MAP decreased below 65 mmHg. Dobutamine requirement were recorded. Cardiac output (Qt) was measured using a lithium dilution technique (LidCo Ltd., Cambridge, UK).

Blood gas analyses (Vetstat, Idexx, France) were performed at 10, 30, and 60 min (T10, T30, T60) after the horse was connected to the machine.

Venous blood was sampled from the jugular catheter into dry tubes 2 h before T0 (T0 − 2), at T0, T5, T10, T15, T30, T45, T60, and 24 h after recovery (TR + 24). This was then stored at −80°C and analyzed 5 months later by gaseous chromatography–mass spectrometry (13) to detect the presence or absence of xenon (detection limit 0.5 nmol/mL).

Creatine kinase (CK) plasma concentration was measured 24 h before the induction of anesthesia (T-24), at TR, TR + 2, and TR + 24. Lactate plasma concentration was measured at T10 and T60 (Vettest, Idexx, France).

Isoprostanes (8-epi PGF_2α_) plasma concentrations were measured in venous blood sampled at T10, T30, T60, and TR + 2 using an AB SCIEX QTRAP 4500 LC-MS/MS System. The method was previously described (14).

## Results

Some results were taken from the Tafonius Data Record that was automatically recorded by the PC during the procedure. Other results, such as from the xenon analyser were obtained from the written anesthesia record as the data were not linked to the Tafonius software.

### Modeling

For the *denitrogenation* phase, calculations predicted that a 1-min denitrogenation period would allow a concentration of 67% oxygen to be reached in the horse’s lungs (Table 1). Oxygen needs only to be supplied at or above maintenance rate during this period as any excess would simply be lost through the ventilator escape valve (dump valve). During the procedure, oxygen was administered at 8 L min^−1^, which is noted by the authors as much in excess of metabolic requirements but did ensure that denitrogenation proceeded as expected.

At the end of the first minute (six breaths), the horse’s FeO_2_ was 72% while our calculations predicted a concentration of 67%. This calculation was based on an FRC of 20 L. Oxygen flow was then stopped and xenon was delivered at 10 L min^−1^.

For the *wash-in* phase, calculations predicted that the most efficient way to reach an expired fraction of xenon (FeXe) of 70% would be to run the xenon flow at 10 L min^−1^. Our predictions also indicated that this would take just over 6 min (38 breaths) and require 63 L of xenon (Table 2).

During the procedure, FeXe was noted to reach 62% after 5 min and 68% after 10 min of continuous xenon flow at 10 L min^−1^ (paper records).

The predicted times were 5 min and 20 s and 6 min, respectively, so the actual time taken, and therefore xenon used, was more than predicted. During these first 10 min, 100 L of xenon was used rather than the predicted 63 L.

The predicted time for FiO_2_ to fall to 30% was 3 min and 40 s from the spreadsheet based on an FRC of 20 L.

During the procedure the wash-in phase required 3 min and 15 s of xenon flow until the FiO_2_ dropped to 30% (from Tafonius SD File), during which time the xenon concentration in the horse rose to 56% (paper records).

For the *maintenance* phase, calculations predicted that this phase would require between 31 and 52 L h^−1^ of xenon (horse’s consumption) to which leaks from the system must be added for 1 h. Oxygen also needs to be added during the maintenance phase to support metabolic demand of the patient. It was anticipated that a 70:30 Xe/O_2_ mix would be supplied during the maintenance phase.

Leakage in the modified anesthetic machine was determined (using the Tafonius electronic leakage calculation mode) to be 1.2 L h^−1^ at peak inspiratory pressures of 30 cm H_2_O and near 0 at 20 cm H_2_O, so only minimal additional loss of xenon was anticipated.

At the start of the maintenance phase, the oxygen was turned on at a flow rate of 5 L min^−1^ for 20 min, and then was reduced to 2–3 L min^−1^ until the end of the procedure to maintain an FiO_2_ around 30%.

Once FeXe had reached 70%, it was maintained between 65 and 70% (65 ± 3%; mean ± SD) with a flow of 0 to 2 L min^−1^ for 30 min [total: 60 L maximum, mean: 30 L (1 L min^−1^ × 30 min)], and then 0 to 3 L min^−1^ for 30 min [total: 90 L maximum, mean: 45 L (1.5 L min^−1^ × 30 min)] during the last 30 min. This meant that we used a maximum of 150 L (mean 75 L) of xenon gas for 1 h of anesthesia (instead of estimated 52 L).

### Total Amount of Xenon Required during the Procedure

The anticipated total amount of xenon to induce and maintain anesthesia for 70 min was, therefore, 63 L plus 52 L = 115 L plus leaks.

As the wash-in phase required 100 L, the maximum total amount of xenon used was 250 L. However, as the flow meter was often set at lower values, it is suspected that the total consumption was probably less with a consumption around 175 L (100 + 30 + 45).

### Depth of Anesthesia

During the procedure, the horse showed five episodes (T25, T35, T45, T60, T70) of nystagmus and/or very slow and slight leg movements, indicating inadequate depth of anesthesia and requiring five additional IV boli of ketamine (0.6 mg kg^−1^) (Table 3), corresponding to a constant rate of 2.5 mg kg^−1^ h^−1^. BIS values were not consistent with these episodes of awareness (84, 61, 61, 74, respectively).

**Table 3:** Physiological data measured over a 70 min period in a horse during general anesthesia with xenon (70%) and oxygen (30%), co-administered with a lidocaine CRI at 0.05 mg kg^−1^ min^−1^.

Time		T0	T5	T10	T15	T20	T25	T30	T35	T40	T45	T50	T55	T60	T65	T70	T-24	TR	TR + 2	TR + 24
HR	(bpm)	23	51	40	36	40	40	40	39	39	56	51	39	37	52	58	–	–	–	–
SAP	(mmHg)	–	–	–	100	95	95	91	100	88	110	94	89	100	83	–	–	–	–	–
MAP	(mmHg)	–	–	–	77	71	70	76	85	69	86	73	71	79	72	–	–	–	–	–
DAP	(mmHg)	–	–	–	61	55	54	51	60	48	67	67	56	64	63	–	–	–	–	–
PE’CO_2_	(mmHg)	37	37	38	39	39	37	39	36	34	38	35	39	37	37	33	–	–	–	–
	(kPa)	4.9	4.9	5.0	5.2	5.2	4.9	5.2	4.8	4.5	5.0	4.6	5.2	4.9	4.9	4.4	–	–	–	–
SpO_2_	(%)	91	87	80	87	85	86	81	85	85	84	86	83	80	81	82	–	–	–	–
BIS		–	–	–	–	–	84	62	61	81	61	41	65	74	–	–	–	–	–	–
FeXe	(%)	0	61.5	67.9	67.9	67.7	65.3	68.6	64.9	69.3	64.6	64.8	65.6	66.1	62.9	59.7	–	–	–	–
FiO_2_	(%)	98	86	24	28	24	28	26	27	23	29	29	29	29	31	35	–	–	–	–
pH		–	–	7.4	–	–	–	7.4	–	–	–	–	–	7.4	–	–	–	–	–	–
PaCO_2_	(mmHg)	–	–	47	–	–	–	47	–	–	–	–	–	51	–	–	–	–	–	–
	(kPa)	–	–	6.3	–	–	–	6.3	–	–	–	–	–	6.8	–	–	–	–	–	–
PaO_2_	(mmHg)	–	–	47	–	–	–	45	–	–	–	–	–	45	–	–	–	–	–	–
	(kPa)	–	–	6.3	–	–	–	6	–	–	–	–	–	6	–	–	–	–	–	–
SaO_2_	(%)	–	–	85	–	–	–	83	–	–	–	–	–	81	–	–	–	–	–	–
HCO3^−^	(mmol L^−1^)	–	–	28.8	–	–	–	29	–	–	–	–	–	30.1	–	–	–	–	–	–
Hb	(g L^−1^)	–	–	11	–	–	–	9.7	–	–	–	–	–	9.8	–	–	–	–	–	–
TP	(g L^−1^)	–	–	58	–	–	–	–	–	–	–	–	–	52	–	–	–	–	–	–
Ht	(%)	–	–	30	–	–	–	–	–	–	–	–	–	27	–	–	–	–	–	–
Lactate	(mmol L^−1^)	–	–	1.3	–	–	–	–	–	–	–	–	–	1.9	–	–	–	–	–	–
Isoprostanes	(ng mL^−1^)	61.2	–	–	–	–	–	23.9	–	–	–	–	–	29.4	–	–	–	–	3.8	–
CK	(mmol L^−1^)	–	–	–	–	–	–	–	–	–	–	–	–	–	–	–	243	301	364	202
Ketamine	(mg kg^−1^)	–	–	–	–	–	0.6	–	0.6	–	0.6	–	–	0.6	–	0.6	–	–	–	–

*HR, heart rate; SAP, systolic arterial pressure; MAP, mean arterial pressure; DAP, diastolic arterial pressure; PE’CO_2_, expired pressure of carbon dioxide; SpO_2_, peripheral oxygen hemoglobin saturation; BIS, bispectral index; FeXe, expired fraction of xenon; FiO_2_, inspired fraction of oxygen. PaCO_2_, arterial partial pressure of carbon dioxide; PaO_2_, arterial blood oxygen tension; SaO_2_, arterial hemoglobin oxygen saturation; HCO3^−^, bicarbonate concentration; Hb, hemoglobin concentration; TP, total protein concentrations; Ht, hematocrit; Lactates, lactate plasma concentrations, CK, creatine kinase plasma concentrations*.

### Cardiorespiratory Variables

Mean (±SD) HR was of 42 ± 9 bpm and mean (±SD) MAP was 75 ± 6 mmHg. Dobutamine was not required. Mean (±SD) P_E_’CO_2_ was 37 ± 1.8 mmHg (4.9 ± 0.2 kPa) and PaCO_2_ (mean ± SD) was 48 ± 2 mmHg (6.3 ± 0.2 kPa).

SpO_2_ was 84 ± 3% and PaO_2_ (mean ± SD) was 45 ± 1 mmHg (5.9 ± 0.1 kPa). All cardiorespiratory data are reported in Table 3.

One single value of Qt was obtained at T35 indicating a cardiac index (CI) of 44 mL kg^−1^ min^−1^.

### Blood Sample Analyses

Blood gas values along with lactate, CK, and isoprostanes plasma concentrations are reported in Table 3.

Xenon serum concentrations could be detected in all samples, except at T0 − 2, T0, and TR + 24.

### Description of Recovery

It was considered a good recovery, taking 35 min, with a single attempt to stand after a brief period (8 min) in sternal recumbency, accompanied by good stability (no stumbling neither striking the recovery stall walls).

## Discussion

These results show that it was easy to adapt the Tafonius machine to deliver xenon although there were some differences between the theoretical calculations and what we observed in practice on a horse. A maximum of 250 L and a mean of 175 L of xenon were required to reach and maintain a 60–70% FeXe in a 500-kg horse for 70 min, using the modified large animal anesthesia machine. However, despite the co-administration of lidocaine and the absence of noxious stimuli, the depth of anesthesia was not sufficient. Low FiO_2_ induced hypoxemia. Recovery was slower than anticipated, composed, and uneventful.

The detection of xenon in all tubes, except before xenon administration and 24 h after recovery, shows its rapid wash-in and wash-out properties. Unfortunately, hemolysis during thawing prevented quantitative analyses.

During the *denitrogenation* period, xenon had to be administered as soon as possible so that arousal could be avoided. For this reason, 100% O_2_ was applied for 1 min only. Calculations anticipated a FeO_2_ of 67% after 1 min. However, experimental data showed that FeO_2_ after 1 min was 72%, suggesting that the horse’s FRC may have been smaller than 20 L, rather around 15 L. By use of the modeling spreadsheet it was determined that an FRC of 15 L would give rise to an FeO_2_ of 72% after six breaths (Table 4) and this may indicate that the FRC of the recumbent horse was less than used in the model.

**Table 4:** Modeling of denitrogenation.

Breath	New horse O_2_%—FeO_2_	New system O_2_%—FiO_2_
1	43.57	87.91
2	56.24	81.70
3	63.51	78.67
**4**	**67.84**	77.37
5	70.57	76.99
6	72.40	77.10
7	73.74	77.47
8	74.81	77.97
9	75.71	78.54
10	76.52	79.13
11	77.27	79.72
12	77.97	80.31
13	78.64	80.89
14	79.28	81.46
15	79.90	82.01
16	80.50	82.54
17	81.09	83.06
18	81.65	83.56
19	82.20	84.05
20	82.73	84.53

*The denitrogenation phase values shows the situation with a machine prefilled with 100% oxygen, a machine’s volume of 28 L and horse’s FRC of 15 L. Calculations predicted that with an oxygen flow at 8 L min^−1^ the FeO_2_ would reach 67% after four breaths*.

We used a spreadsheet model to anticipate the FeO_2_. The use of differential equation describing the balance between inflow and outflow might have been more accurate. Thanks to these equations, Bukoski showed that the accuracy of wash-in kinetics of oxygen into a large animal circle breathing system depends on the time to 50% oxygen concentration, the initial and target concentration (15).

The *wash-in* period was surprisingly rapid. It involved equilibrating the machine (cylinder and circuit) and the horse’s FRC at a 70% concentration of xenon. The spreadsheet predicts that xenon (FeXe) would reach 70% in 38 breaths, or 6 min and 20 s, and oxygen (FiO_2_) would reach 30% after 22 breaths, or 3 min and 40 s. However, as the horse’s FRC may have been 15 L rather than 20 L, modifications of the FRC on the spreadsheet show that 30% oxygen (FiO_2_) would be reached after 20 breaths or 3 min and 20 s and 70% xenon (FeXe) would be achieved after 34 breaths, or 5 min and 40 s (Table 5), which is in accordance with experimental data from Tafonius and the written anesthesia record.

**Table 5:** Modeling of wash-in phase.

Breath	Horse Xe%—FeXe	Cumulative Xe volume required (L)	Horse O_2_%—FeO_2_	System O_2_%—FiO_2_
1	0.00	1.67	67.00	63.01
2	1.70	3.33	65.86	59.87
3	4.27	5.00	64.15	57.22
4	7.25	6.67	62.17	54.88
5	10.41	8.33	60.09	52.73
6	13.59	10.00	57.98	50.72
7	16.75	11.67	55.91	48.81
8	19.83	13.33	53.88	46.99
9	22.81	15.00	51.91	45.25
10	25.71	16.67	50.01	43.57
11	28.49	18.33	48.17	41.97
12	31.18	20.00	46.40	40.42
13	33.77	21.67	44.69	38.93
14	36.26	23.33	43.04	37.49
15	38.66	25.00	41.46	36.11
16	40.97	26.67	39.93	34.78
17	43.20	28.33	38.46	33.50
18	45.34	30.00	37.04	32.26
19	47.40	31.67	35.67	31.07
**20**	49.38	33.33	34.36	**29.93**
21	51.28	35.00	33.09	28.82
22	53.12	36.67	31.87	27.76
23	54.88	38.33	30.70	26.74
24	56.58	40.00	29.57	25.75
25	58.22	41.67	28.48	24.80
26	59.79	43.33	27.43	23.89
27	61.31	45.00	26.42	23.01
28	62.76	46.67	25.44	22.16
29	64.17	48.33	24.51	21.35
30	65.52	50.00	23.60	20.56
31	66.82	51.67	22.73	19.80
32	68.07	53.33	21.90	19.07
33	69.27	55.00	21.09	18.37
**34**	**70.43**	56.67	20.31	17.69
35	71.54	58.33	19.56	17.04
36	72.61	60.00	18.84	16.41
37	73.64	61.67	18.15	15.81
38	74.64	63.33	17.48	15.22
39	75.59	65.00	16.83	14.66
40	76.51	66.67	16.21	14.12

*Immediately after the denitrogenation phase, the spreadsheet starts with a FeO_2_ of 67% and a xenon flow of 10 L min^−1^. Calculations used to model gas movements, considering a FRC of 15 L, a tidal volume of 6 L, and *f*_R_ of six breaths min^−1^, indicated that FiO_2_ reached 30% after 20 breaths (3 min and 20 s) and FeXe achieved 70% after 34 breaths (5 min and 40 s)*.

The volume of FRC used for the calculation was measured in awake horses (10). It is well known that FRC decreases when the horse is anesthetized (16) due to the anesthetic drugs effects and the recumbency. The modification of the diaphragmatic outline, consequently to positional changes, is the cause of the decrease in FRC (17). In addition, xenon has the potential to cause expansion of closed, gas filled spaces. Therefore, the cecum volume and the intraabdominal pressure may have increased during anesthesia. This could have exerted additional pressure on the diaphragm and could have further decreased the FRC. This highlights the difficulty in modeling the horse and machine characteristics when there could be a large variation in size of the FRC.

The spreadsheet proved to be rather useful to anticipate gas movements, but could be further refined to model more accurately xenon and oxygen uptake. For example, we did not take into account the horse Xe consumption to model the wash-in phase. It can be shown that modeling xenon uptake at 52 L per hour (0.1 L kg^−1^ h^−1^) would change the predicted arrival at a FeXe of 70% by an additional 2–3 breath. To take Xe consumption into account was not considered necessary for two reasons: the actual uptake rate by the horse was unknown and the percentage error is small compared with 25–30% changes in predicted FRC.

The wash-in phase could be made more efficient and use less xenon if the expired xenon could be recycled and not lost from the system. The minimum amount of xenon required to achieve 70% concentration in a 48-L system is 34 L but this assumes that all added xenon replaces system oxygen with no loss of xenon. Xenon is lost when mixed expired gas is voided from the system through the spill valve. Reducing oxygen delivery during the wash-in phase to the minimum required to maintain a FiO_2_ of 30% would improve efficiency as in the initial period oxygen in the system would be consumed by the horse and in the subsequent period only metabolic demand needs to be supplied. This would reduce the volume added to the system and, therefore, reduce the amount of gas lost through the spill valve and hence loss of xenon. This and other strategies could be investigated in further studies; however, for this first attempt, at xenon usage in the horse such fine-tuning may have over-complicated the procedure.

During the *maintenance phase*, the xenon requirements come from consumption by the patient and from leaks in the system. The consumption of xenon in humans is known to be 0.06–0.1 L kg^−1^ h^−1^ (12). We assumed that the horse’s consumption rate would be similar, and therefore, it was expected to reach 52 L h^−1^, to which leaks from the system (1.2 L h^−1^) should be added. Unfortunately, because the regulations of xenon and oxygen flows were continuously requiring small manual changes, it was impossible to record and accurately calculate the xenon consumption and only the assumption of a maximum of 150 L h^−1^ for a horse weighting 514 kg can be made. In order to be more accurate and avoid wastage, several precautions will have to be taken for further experiments.

First, setting up an automated system based on a target of 30% oxygen would greatly improve the gas efficiency of the system, and make it more comfortable for the anesthetist. A study compared xenon consumption of different delivery modes (ECO mode, AUTO mode, or MANUAL mode) provided by the closed-circuit respirator Felix Dual ND during general surgery in human patients (12). It revealed significant reduced xenon consumption during the automatic modes in comparison to the manual mode.

Second, as no emergency high flows of xenon were required, a 15-L min^−1^ flow meter could have been used, instead of a 50 L min^−1^ flow meter. This would have improved the accuracy of the values at low flow rates. Even if it is of a minor effect here, it can be added that there is a tolerance of ±4% on the reading of the flow meter meaning it is possible that we used an extra 4%, or 10 L. The flow meter could also be calibrated to 5 bars at 15°C, so it displays the flow rate at 15°C. Unfortunately, for this experiment, the flow meter and the regulator were calibrated to 4 bars at 20°C, which could have led to an overpressure and an increased consumption of 33%, meaning that we have possibly used up to 250 L × 1.33 = 333 L. The use of a 15-L min^−1^ electronic mass flow meter seems to be a more suitable solution for further studies. As a 30°C temperature difference could also change the viscosity of xenon by 10%, it is important to assess the room temperature.

Furthermore, we could have weighted the xenon cylinder before and after the trial to estimate the volume of xenon consumed.

In summary, our greatest difficulty was to accurately calculate the xenon volumes used during the clinical phase of this study. Logging of the xenon concentrations and flow would have proved invaluable. These are not standard features of the xenon analyzer and additional equipment should be designed to allow this.

The horse presented five episodes of nystagmus and/or slow leg movements, requiring top-ups of ketamine. This did not seem to coincide with any particular fluctuations in expired xenon content. Although it is difficult to assume it in only one horse the MAC of xenon in horses might be higher than 70%. In man, FeXe 70% is often associated with the administration of a potent μ agonist with sparing anesthetic effect, which leads us to think that the MAC might be different (2). Lidocaine volatile anesthetic-sparing effect in horses is well recognized (18, 19). In order to imitate the sparing effect of fentanyl, we added lidocaine in our anesthetic protocol. The total dose of additional boli of ketamine over time corresponded to an infusion rate of 2.5 mg kg^−1^ h^−1^ of ketamine. This dose cannot be considered enough to maintain anesthesia by itself (20). Nevertheless, no noxious stimuli were applied during the procedure suggesting that the depth of anesthesia needs to be improved during painful surgeries. It is also worth highlighting that the xenon analyzer used also measures CO_2_ levels in expired gases. As passing 100% CO_2_ through the analyzer gives a reading of 28% on the screen, the presence of 5% of CO_2_ during the trial could have led to a false elevation of measured FeXe by 1.4% assuming a linear CO_2_ response. This means that a concentration of 70% may not have been fully reached (68.6% would be the true concentration), which may possibly account for the altered quality of narcosis.

The interest of the BIS to monitor the depth of anesthesia in horses is controversial. Previous experiment using isoflurane (21), propofol (22), sevoflurane, or halothane (23) showed the poor quality of BIS to accurately anticipate movements during surgery in horses. Nevertheless, a recent study demonstrated that the BIS value could be useful as an indicator of awakening during the recovery period in horses (24). In our study, BIS values did not allow to anticipate episodes of awareness.

It is difficult to conclude that xenon was responsible for the steady and well-maintained cardiovascular parameters as it was co-administered with lidocaine and ketamine, which may have had some effects on HR and blood pressure.

Repeated Qt measurements were not available, the only measurement obtained at T35 (Qt: 22 L min^−1^; CI: 44 mL kg^−1^ min^−1^) was moderately low compared to the CI obtained from horses anesthetized with isoflurane 45 min after induction (50.73 ± 14.77 mL kg^−1^ min^−1^) (25). Lithium dilution accuracy can be positively biased by the IV administration of drugs, such as lidocaine and ketamine (26), so the value we recorded might not be representative of the real Qt.

Hypoxemia was revealed by PaO_2_ and SpO_2_ values, while lactate and CK plasma concentrations remained within the normal range.

The administration of 70% xenon requires reducing the inspired fraction of oxygen to 30%. An FiO_2_ of 30–35% was enough to maintain normal PaO_2_ in humans during anesthesia (27) and hypoxemia was not a concern when xenon was administered to healthy human patients or animals in which it has been already tested, for example in pigs that breathed 70% xenon and 30% O_2_ (28). For these reasons, we did not anticipate that a major hypoxemia would occur and low values of PaO_2_ were not considered as end points in this study.

However, hypoxemia is a frequent complication during equine general anesthesia with drugs labeled for use in horses and a study reports that an FiO_2_ of 30% increased the risk for intraoperative hypoxemia and failed to improve postoperative arterial oxygenation in healthy horses (29). In horses breathing 21% O_2_ under IPPV, PaO_2_ decreased to 60 ± 10 mmHg after 2 h of anesthesia (30).

Nevertheless, although it is important to prevent hypoxemia (as its management is often unsuccessful in horses), there is no evidence that it is associated with increased morbidity and mortality in anesthetized horses (31). Hypoxemia occurring under general anesthesia is harmful when lactate is produced and accumulates within tissues. Acidosis adversely affects metabolic, mechanical, and electrical cells activities. This can contribute to altered cognition function, fall in cardiac output, and acute respiratory distress syndrome in human patients. This has not been proven in horses and may be because the affinity of hemoglobin for oxygen is greater in horses compared with that in humans (31). In our study, oxygen hemoglobin saturation values were between 80 and 90%. Lactate plasma concentration did not increase, the procedure was short and the horse was healthy. For these reasons, we decided to tolerate this complication and to complete the study.

One available reference describing the consequence of hypoxemia in horses shows that muscle oxygenation is reduced (30) and this could affect the ability of the horse to stand during recovery. Although we have obtained even lower PaO_2_ values than previously reported (29, 30), we have no strong evidence that hypoxia was present and that it did affect tissue metabolism and perfusion. The recovery was uneventful and the horse did not show signs of cognitive dysfunction. However, these results are important to take into account in order to anticipate this complication during longer surgery on a sick horse.

The recovery was considered slow and steady. This observation was supported by CK measurements during and after the general anesthesia, which did not increase from the basal value. The recovery time was unexpectedly slow considering the low blood/gas partition coefficient of xenon. This may result from the additional anesthetic drugs given during the procedure and just before recovery.

F_2_ isoprostanes, especially 8-epi-PGF_2α_, are specifically produced from arachidonic acid by a non-cyclooxygenase-mediated peroxidation pathway which involves the auto-oxidation of polyunsaturated fatty acids (14). They can be found in plasma, tissue, urine, and bronchoalveolar fluid following oxidant exposure in humans and animals (32). Quantification of F_2_ isoprostanes seems to be the earliest and most accurate method currently available to assess oxidative stress *in vivo*. In this study, the concentrations observed were more than ten times higher than in a study conducted in horses anesthetized with isoflurane under 100 or 21% oxygen (30) and compared to a study conducted in horses undergoing exercise (33). We assume that oxidative stress may have occurred at the time of thawing (34).

Anti-inflammatory (35) and antioxidative (36) properties of xenon are now well known. In the study by Portier et al. (30) volatile anesthesia associated to hyper or hypoxemia did not induce significant changes in isoprostanes plasma concentrations. In our study, lipid oxidative stress assessed by isoprostanes concentrations decreased over time. This could be due to xenon anesthesia-antioxidative properties. However, our procedure was short and minimally invasive, for which halogenated agents showed similar benefits (37). It would be worthwhile to explore this property in the systematically ill patient, or for longer and more invasive procedures, for which it is known that volatile agents increase oxidative stress and inflammation (37).

The xenon price varies according to the law of supply and demand. If we consider a cost of about 10 euros per liter, the cost of 70 min of anesthesia according to our study would be between 1,750 and 2,500 euros. Other techniques must be developed, such as recycling, increasing flow meter accuracy, and volume control to reduce the amount of xenon needed to anesthetize an adult horse. With advance scavenging and low-flow techniques it is conceivable that xenon anesthesia could be only slightly more expensive than volatile anesthesia (38). The major problem remains the availability of the gas. Indeed, if we consider that the ceiling on xenon production is around 120 million liters and that the number of major surgeries is estimated as 234 million cases per year in human medicine (requiring around 36 L per case), only 0.5% of all anesthetics could be performed using xenon before the supply ceiling is exceeded (38). Therefore, its potential availability for use in veterinary medicine is questionable.

In conclusion, this pilot study describes how to deliver xenon to a horse. The spreadsheet calculations allowed some predictions of the required flows and timings. It was easy to adapt the Tafonius machine to deliver the xenon; however, some technical problems were encountered. Their correction could guide future endeavors to study the use of xenon in horses.

## Ethics Statement

The study was performed in accordance with the EUROGUIDE on the accommodation and care of animals used for experimental and other scientific purposes published by the Royal Society of Medicine Press Limited (London, UK). The experimental procedure was approved by the Animal Ethics Committee of VetAgro-Sup (veterinary campus of Lyon), France (no. 1535).

## Author Contributions

Participated in research design: BS, KS, AR, MG, JP, and KP. Conducted experiments: BS, KS, AR, JP, and KP. Performed data analysis: KS, AR, MG, and KP. Wrote or contributed to the writing of the manuscript and revised the manuscript: BS, KS, AR, MG, and KP.

## Conflict of Interest Statement

KS is the managing director of Vetronic Services Limited, who developed the modification of the equine anesthesia station for delivering xenon. The authors declare that this study received funding from Messer Group GmbH for providing xenon. The funder was not involved in the study design or collection, analysis, or interpretation of the data.
